# Pectinolytic lyases: a comprehensive review of sources, category, property, structure, and catalytic mechanism of pectate lyases and pectin lyases

**DOI:** 10.1186/s40643-021-00432-z

**Published:** 2021-08-23

**Authors:** Ling Zheng, Yinxiao Xu, Qian Li, Benwei Zhu

**Affiliations:** College of Food Science and Light Industry, Nanjing Tech University, Nanjing, 211816 P. R. China

**Keywords:** Pectin lyase, Pectate lyase, Sequence analysis, Biochemical properties, Structure, Catalytic mechanism

## Abstract

Pectate lyases and pectin lyases have essential roles in various biotechnological applications, such as textile industry, paper making, pectic wastewater pretreatment, juice clarification and oil extraction. They can effectively cleave the α-1,4-glycosidic bond of pectin molecules back bone by β-elimination reaction to produce pectin oligosaccharides. In this way, it will not generate highly toxic methanol and has the advantages of good enzymatic selectivity, less by-products, mild reaction conditions and high efficiency. However, numerous researches have been done for several decades; there are still no comprehensive reviews to summarize the recent advances of pectate lyases and pectin lyases. This review tries to fill this gap by providing all relevant information, including the substrate, origin, biochemical properties, sequence analysis, mode of action, the three-dimensional structure and catalytic mechanism.

**Supplementary Information:**

The online version contains supplementary material available at 10.1186/s40643-021-00432-z.

## Key points

- Provided the information about pectate lyases and pectin lyase involving substrate, source, biochemical properties, mode of action and sequence.
- Summarized the three-dimensional structure and catalytic mechanism of pectate lyases and pectin lyases comprehensively.

## Introduction

Pectin is a structural polysaccharide that forms the component of the primary cell wall and middle lamella of plants in fruits and vegetables (Kohli and Gupta 2015; Wu et al. 2020). Due to its rigidity and flexibility, pectin can protect the plants from invasion of pathogenic microorganism’s and mechanical injury. Thus, plant pathogens attack target cells by producing cell wall-degrading enzymes, including pectinases, cellulases and proteases. Pectinase can degrade pectin into oligomeric products with various physiological activities by physical degradation, chemical hydrolysis, and enzymatic preparation (Chen et al. 2013; Gómez et al. 2014; Kang et al. 2006; Tobias et al. 2017; Wang et al. 2019). Enzymatic reactions have obvious advantages such as mild reaction conditions, high specificity and degradation efficiency compared with physical and chemical methods. Therefore, the enzymatic preparation of pectin oligosaccharides (POSs) with good biological activity has obtained increasing attention. POSs could be selectively utilized by the intestinal microorganisms and were considered as the best choice for second-generation prebiotic factors (Olano-martin et al. 2020). Moreover, POS exhibited various physiological activities, such as prebiotic, antibacterial, anticancer and antioxidant properties, which could be developed as functional food additives (Zhu et al. 2019). For example, Kang et al*.* produced pectin-oligosaccharide from citrus by irradiation and found that POS exhibited antioxidant and anticancer effects (Kang et al. 2006). Li et al. depolymerized the orange peel polysaccharide into POS enzymatically, and confirmed that POS have prebiotic properties and antibacterial activity (Li et al. 2016).

Of all pectinases, pectin lyase and pectate lyase have attracted great attention. Both can cleave the α-1, 4-glycosidic bonds by β-elimination to generate 4,5-unsaturated oligogalacturonides without producing highly toxic methanol (Saharan and Sharma 2019; Sassi et al. 2017). Pectin lyase and pectate lyase are also known as the polymethylgalacturonate lyase (PMGL) and polygalacturonate lyase (PGL), respectively (Jayani et al. 2005). According to the amino acid sequence, PGLs are classified into polysaccharide lyase (PL) families 1, 2, 3, 9 and 10 (Kamijo et al. 2019; Zhou et al. 2017). Based on carbohydrate active enzymes (CAZy) database (http://www.cazy.org/), PMGLs (EC 4.2.2.10) only exist in PL1 family (Vincent et al. 2013). In terms of three-dimensional structure, the current data of CAZy database shows that the PGLs of PL1, 3, and 9 families have parallel β-helix structure, the PGLs of PL2 family display (α/α)_7_-barrel structure, and the PGLs of PL10 family exhibit (α/α)_3_-barrel structure. Notably, only two structures of PMGLs from PL1 family have been solved (PDB: 1IDJ and 1QCX), which show β-helix structure.

So far, numerous related researches have been reported (Damak et al. 2011; Kashyap et al. 2001; Liang et al. 2014; Yadav et al. 2008). However, there are still no comprehensive reviews to summarize the recent advances of PGLs and PMGLs. Therefore, this review aimed to clarify the origin, classification, sequence analysis, mode of action, three-dimensional structure, and catalytic mechanism of PGLs and PMGLs. Since pectin is the substrate, it is essential to discuss the salient features of its structure and property in brief. This work is of great significance to promote our understanding of these pectin-degrading enzymes and expand the applications of these enzymes in food and industrial fields.

## Pectin: origin, structure, and property

According to the American Chemical Society (ACS), pectic substances are divided into four main types, namely protopectin, pectic acid, pectinic acid and pectin (Jayani et al. 2005). Among them, pectin is a macromolecular polysaccharide with complex structure. It widely exists in the cell walls of some fruits and vegetables, such as citrus, lemon, apple, and pumpkin (Vasco-Correa and Zapata Zapata 2017). Pectin from various sources differs in the degrees of esterification (DE). Generally, pectin is classified as high methoxy pectin (DE > 50%) and low-methoxy pectin (DE < 50%) (Bermúdez-Oria et al. 2018). As the by-products of juice production in the food industry, the peel and waste residues of citrus, apple, and pomelo are significant raw materials for pectin manufacturing.

**Fig. 1:**
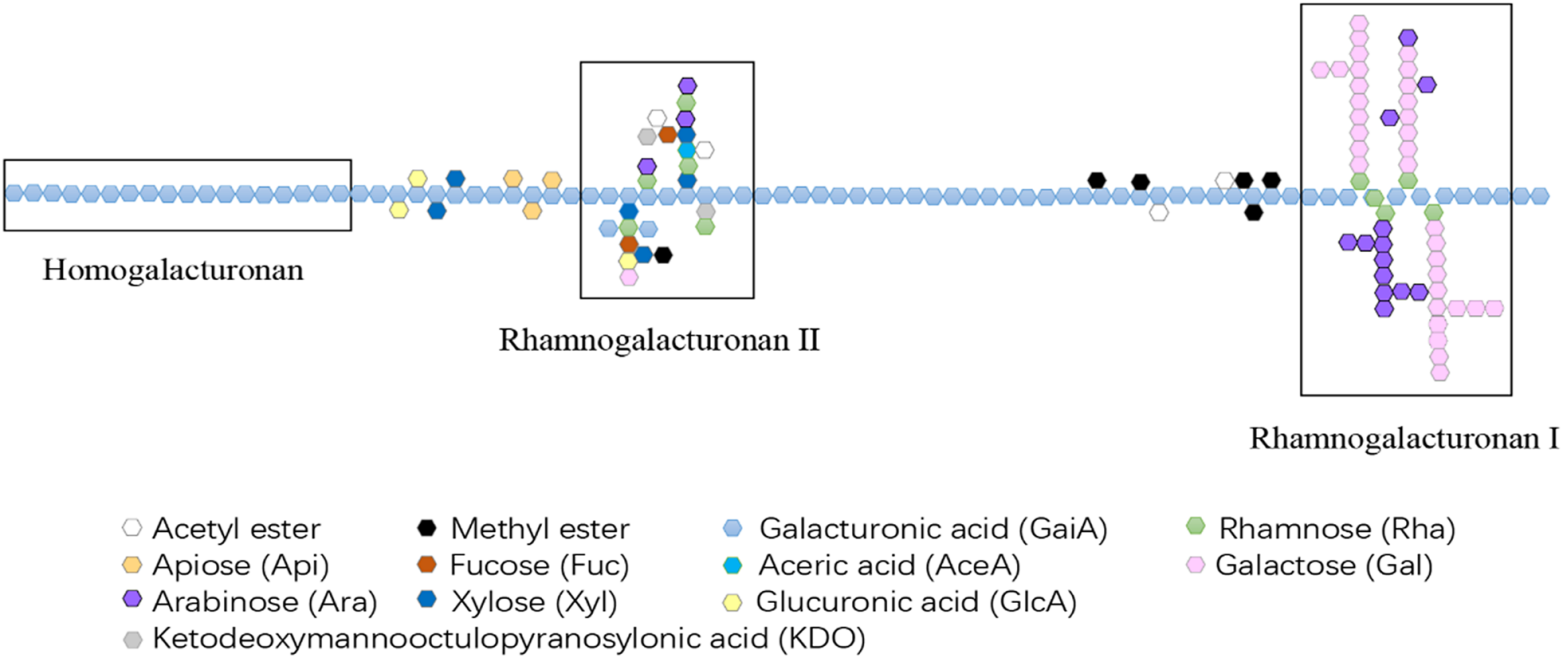
The structure and composition of pectin

Pectin is acidic heteropolysaccharide linked by D-galacturonic acid via α-1,4-glycosidic bonds (C. Zhou et al. 2017a, b). Its side chain is mainly composed of rhamnose, arabinose, galactose, and xylose (Kohli and Gupta 2015). Generally, the natural pectin is not linear, but displays a multi-branched structure. Thereby, it can be divided into smooth region and hairy region (Sinitsyna et al. 2007). As shown in Fig. 1, it is mostly composed of three structurally well-characterized polysaccharide motifs, the homogalacturonan (HGA), the rhamnogalacturonan I and rhamnogalacturonan II (RGI and RGII), and a small part of xylogalacturonan (XGA) (Jayani et al. 2005).

Pectin differs in composition as well as properties from diverse sources. The degree of methyl esterification usually has a significant impact on the property of pectin. It becomes a viscous colloidal solution in water, and is insoluble in mostly organic solvents. Pectin can undergo ester hydrolysis or breakage of glycosidic bonds to generate galacturonic acid and alcohol in the presence of acid, alkali, or pectinase. Moreover, it can form gel with sugar, acid or calcium ions under proper conditions, such as high sugar concentration and low pH conditions (Kohli and Gupta 2015). The low-methoxy pectin forms gel even without sugar in the presence of calcium ions (Vasco-Correa and Zapata Zapata 2017). The gel-forming ability of pectin has been widely used in food and pharmaceutical industries as thickening agent, stabilizer and emulsifier in food processing (Pérez et al. 2000). Pectin also plays a vital role in medicine, cosmetics, and other industries, such as the production of health products for the prevention and treatment of diabetes and obesity.

## Source, category and application of pectin and pectate lyase

Pectinases are a group of enzymes involved in pectin degradation (Saharan and Sharma 2019) (Table 1). The complete degradation of pectin requires the synergistic effect of different pectinases with diverse substrate specificity (Kamijo et al. 2019; Kikuchi et al. 2006). There are various classification criteria for pectinase. According to the optimal pH, it can be divided into alkaline and acid pectinase. Based on the substrate and mode of action, pectinases are mainly classified into pectin esterase, pectin hydrolase, propectinase, pectin lyase and pectate lyase as shown in Table 1 (Jayani et al. 2005; Kashyap et al. 2001).

**Table 1:** Classification of pectinases

Enzyme			E.C. no	Substrate	Reaction type	Products
PE	PME		EC3.1.1.11	Pectin	Deesterification	Pectic acid + methanol
	PAE		EC3.1.1.6	Pectin	Deesterification	Pectic acid methanol
Depolymerases	Hydrolases	Endo-PG	EC3.2.1.15	Pectic acid	Hydrolysis	Oligogalacturonates
		Exo-PG1	EC3.2.1.67	Pectic acid	Hydrolysis	Monogalacturonates
		Exo-PG2	EC3.2.1.82	Pectic acid	Hydrolysis	Digalacturonates
	Lyase	Endo-PGL	EC4.2.2.2	Pectic acid	Trans-elimination	Unsaturated oligogalacturonates
		Exo-PGL	EC4.2.2.9	Pectic acid	Trans-elimination	Unsaturated digalacturonates
		Endo-PMGL	EC4.2.2.10	Pectin	Trans-elimination	Unsaturated methyloligogalacturonates
PPase			–	Protopectin	Hydrolysis	Pectin

*PE* pectin esterase, *PME* pectin methyl esterase, *PAE* pectin acetyl esterase, *PG* polygalacturonase, *PGL* polygalacturonate lyase, *PMGL* polymethylgalacturonate lyase, *PPase* protopectinase

Pectin lyase (PMGL) and pectate lyase (PGL) can directly cleave the α-1,4-glycosidic bond of the pectin molecular skeleton by β-elimination mechanism without producing highly toxic methanol, which has obtained increasing attention (Jayani et al. 2005). They are known as polymethylgalacturonic acid lyase (PMGL) and polygalacturonic acid lyase (PGL), respectively. Generally, most of PGLs and PMGLs are derived from microorganisms such as bacteria and fungi, only a few of enzymes originate from plants and animals (Table 2). For instance, Yadav et al. purified and characterized an alkaline pectin lyase from *Aspergillus flavus* (S. Yadav et al., 2008a, b). Dong et al. isolated two pectate lyases from *Fusarium oxysporum* (Dong et al. 2011)*.* Jenkins et al. analyzed the crystal structure of the pectate lyase Pel9A from *Erwinia chrysanthemum* (Jenkins et al. 2004).

**Table 2:** Biochemical properties of PGLs and PMGLs

Source	PL	Category	Name	M.W. (kDa)	Temperature (^o^C)	pH	Cations	Products	Reference
*Bacillus sp.* N16-5	PL1	PGL	Bsp165PelA	42	50	11.5	–	DP2,3	Gang et al. (2010)
*Aspergillus luchuensis* var. saitoi	PL1	PGL	AsPelA	35	30	8.0	Ca^2+^	DP3,4	Kamijo et al. (2019)
*Aspergillus parasiticus*	PL1	PGL	PelB	44	55	9.1	Ca^2+^	DP2-4	Wang et al. (2014)
*Bacillus licheniformis*	PL1	PGL	BliPelA	35	70	11.0	Ca^2+^	DP2,3	Zhou t al. (2017a, b)
*Yersinia enterocolitica*	PL2	PGL	YePL2A	–	–	9.6	Mn^2+^/Ni^2+^	DP2-5	Abbott and Boraston (2007)
*Erwinia carotovora*Ec153	PL2	PGL	Pel153	56	–	–	Ca^2+^	–	Trollinger et al. (1989)
*Paenibacillus amylolyticus*	PL3	PGL	PelB	–	55	9.5	Ca^2+^	–	Boland et al. (2010)
*Bursaphelenchus xylophilus*	PL3	PGL	rBxPEL1	27	55	8.0	Ca^2+^	-	Kikuchi et al. (2006)
*Bursaphelenchus xylophilus*	PL3	PGL	BxPEL3	–	–	9.0	Ca^2+^	–	Lee et al. (2013)
*Bacillus* sp. Strain KSM-P15	PL9	PGL	Pel-15H	70	55	11.5	Ca^2+^	–	Ogawa et al. (2000)
*Xanthomonas campestris*	PL10	PGL	r-PL D	38	30	9.0	Ca^2+^	–	Yuan et al. (2012)
*Bacillus alcalophilus *NTT33	PL10	PGL	Pel-A	35	45	9.5	Ca^2+^	–	Zhai et al. (2003)
*Penicillium occitanis*	–	PGL	Pel1	39	60	9.0	Ca^2+^	–	Damak et al. (2011)
*Aspergillus parasiticus*	–	PGL	ApPel1	41	50	4.0	Ca^2+^	DP2-5	Yang et al. (2020)
*Aspergillus terricola *MTCC 7588	–	PMGL	PNL	35 ± 1	50	8.0	–	–	Yadav et al. (2009a)
*Paenibacillusamylolyticus *CCT7128	–	PMGL	PL	–	40	7.9	–	–	Sakiyama et al. (2010)
*Rhizopus oryzae*	–	PMGL	PL	31	50	7.5	–	–	Hamdy (2005)
*Aspergillus ficuum *MTCC 7591	–	PMGL	PNL	31.6	50	5.0	–	–	Yadav et al. (2008a, b)

Both PGLs and PMGLs belong to pectinolytic lyases, which are also called pectic transeliminases. However, PGLs and PMGLs have obvious differences in their substrate specificities. To be specific, PGLs act on low methyl esterified pectic acid to generate oligogalacturonic acid or digalacturonic acid, while PMGLs catalyze highly methylated pectin to obtain methylated oligogalacturonic acid (Lima et al. 2017). PMGL only degrades the substrate in an endo-type manner, PGL exhibited endo- or exo-modes of action (Bekli et al. 2019). Therefore, they are further subdivided into three categories based on the substrate specificity and the mode of action, namely the endo-polygalacturonate lyase (endo-PGL), the exo-polygalacturonate lyase (Exo-PGL) and the endo-polymethylgalacturonate lyase (endo-PMGL) (Table1).

PGLs and PMGLs have essential roles in various biotechnological applications, which have been extensively investigated (Kohli and Gupta 2015; S. Yadav 2009b). Generally, textile and paper pulp industrial processing are running in alkaline conditions, beverage processing works in acidic conditions (Wu et al. 2020). As shown in Table 2, most PGLs presented the optimum pH from 8 to 11.5 except for PGL from *Aspergillus parasiticus* (ApPel1). Thus, alkaline PGLs are mainly applied in the textile industry presently. For example, Yadav et al. purified and characterized a pectin lyase from *Aspergillus terricola* and clarified its application in retting of natural fibers (S. Yadav 2009a). PGLs also have potential applications in paper making, pectic wastewater pretreatment, coffee and tea fermentation, and oil extraction (Yuan et al. 2012). In addition, PMGLs are commonly used in the beverage industry for juice extraction and clarification, as this process is operated under acidic condition (Sharma et al. 2016). For instance, Yadav et al. purified and characterized an acidic pectin lyase from *Aspergillus ficuum* strain MTCC 7591 for clarification of fruit juices (S. Yadav 2008a). Therefore, PGLs and PMGLs are of great importance in the current biotechnological era with their embracing applications.

## Sequence analysis of PGLs from different PL families

**Fig. 2:**
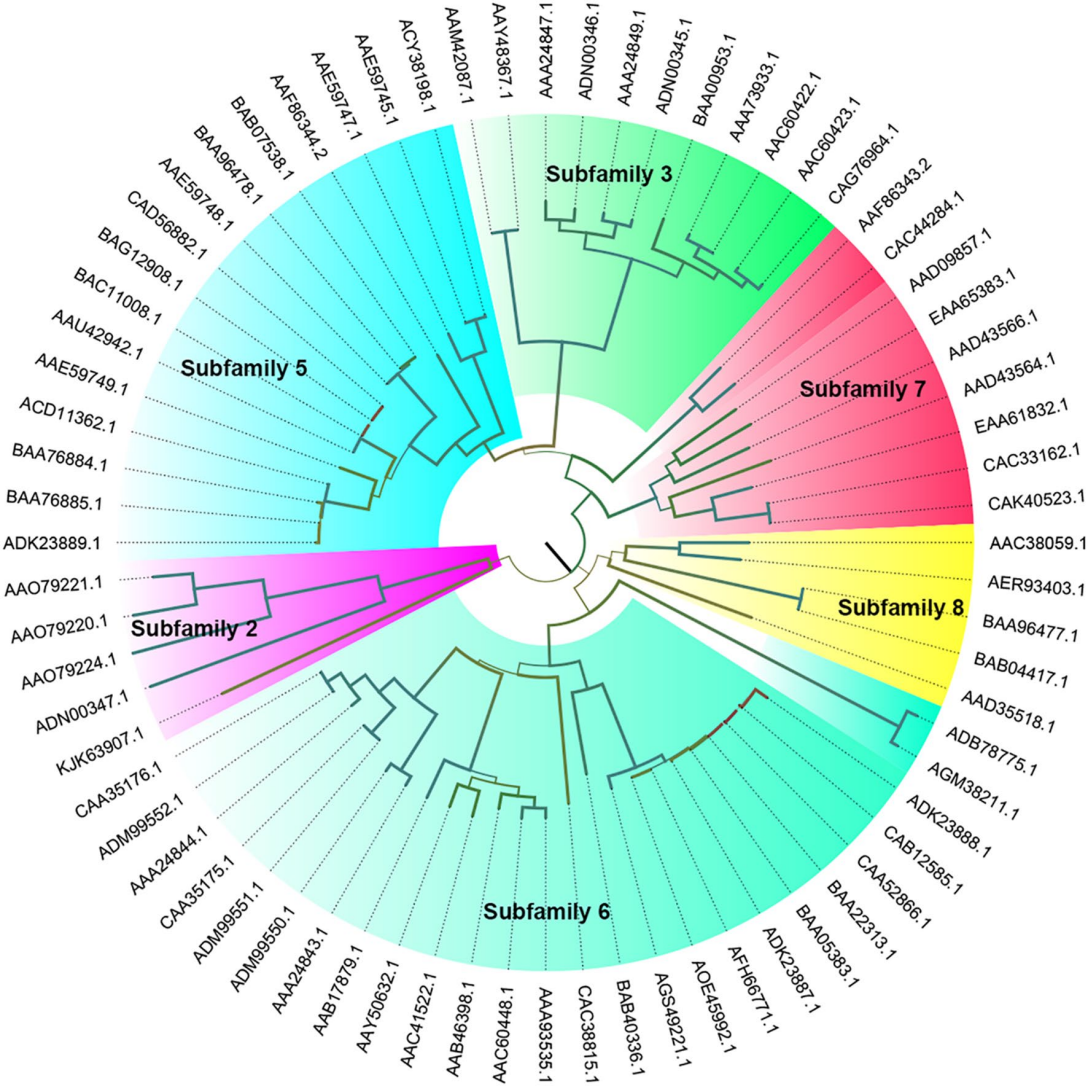
Phylogenetic analysis of PGLs from the PL1families. Different color blocks represented different subfamilies of PL1 family, respectively. Light purple: subfamily 2, bright green: subfamily 3, bright blue: subfamily 5, celeste: subfamily 6, yellow: subfamily 7

According to the amino acid sequence, PGLs are classified into polysaccharide lyase (PL) families 1, 2, 3, 9, and 10 (Heffron et al. 1995). Based on the Carbohydrate Active Enzymes (CAZy) database (http://www.cazy.org/), PMGLs (EC 4.2.2.10) only exist in PL1 family (Vincent et al. 2013). At present, the PGLs from bacteria and fungi have been widely reported. To determine the sequence identity and key amino acid residues, we performed alignment analysis of PGLs from PL 1, 2, 3, 9 and 10 families, respectively (Additional File 1: Figure S1–5). Moreover, PL1 family has the most PGLs characterized and structure-solved. Thus, we constructed a phylogenetic tree to analyze the evolutionary process and subfamily of PGLs in the PL 1 family (Fig. 2). The total sequence identity of the same PGL subfamily is generally higher than 50%, but the sequence identity between subfamilies is much lower. Additionally, some proteins have low sequence identity, but they have similar structures, indicating that proteins with certain sequence characteristics may have analogous structures. For instance, PelC from *Erwinia chrysanthemi* (Yoder et al. 1993), PelE from *Erwinia chrysanfhem* (Lietzke et al. 1996) and Pel from *Bacillus subtilis* (Pickersgill et al. 1994) do not have high sequence identity, but they all have similar β-helix structures. In addition to the same fold, the enzymes share other unique structural features, including a highly organized core consisting of linear arrays of a11 side chains that are oriented toward the interior. Perhaps they shared some specific sequence features, but more research is needed to prove it.

In the PL1 family, PGLs AAE59748.1 and BAB07538.1 (GenBank number) have 98% sequence identity. Based on the structure of CAB12585.1 (PDB:1BN8) from *Bacillus subtilis* (Pickersgill et al. 1994), Asp223 and Asp227 are closely related to the binding of primary calcium. Furthermore, many amino acids involved in substrate binding are conserved, such as Lys247 and Arg284. Interestingly, these four amino acids are highly conserved in the PL1 family. They correspond to Asp244, Asp248, Lys268 and Arg305 of AAE59748.1, BAB07538.1, CAD56882.1 and BAB04417.1 in this review, respectively (Additional file 1: Figure S1).

In the PL2 family, only two PGLs have been settled in structure, five PGLs and one PMGL have been characterized. Sequence comparison revealed that the known PGLs from PL2 family have high sequence identity, ranging from 78 to 99%. By analyzing the structure of YePL2A (PDB: 2V8I) from *Yersinia enterocolitica*, we found that Arg171 and Arg272 at the + 1 subsite are amino acids involved in catalysis, Glu130, Lys132 and Glu515 at the + 1 subsite are closely related to the binding of polysaccharide substrates. As shown in Additional file 1: Figure S2, the above five amino acids (Arg194, Arg295, Glu153, Lys155 and Glu538) are highly conserved in the known sequences. Furthermore, the metal binding pocket of YePL2A mainly includes His109, His172 and Glu130 (corresponding to His132, His195 and Glu153 in this article), which are conservative in the PL2 family. The above-mentioned conserved amino acid residues also confirm that proteins with certain sequence characteristics may have similar structures.

Compared with PL1 and 2 families, PGLs from PL3 family has lower sequence identity. For example, ADB78744.1 and BAA87892.1 have the highest identity of 78%. AAA57140.1 and ADM99140.1 share 65% sequence identity, and ADM99410.1 and ACM60942.1 possess the lowest sequence identity of 21%. With BAA87892.1 (PDB: 1EE6) as a template, sequence alignments revealed some conserved residues: Asp_63_, Glu_83_ and Asp_84_ are related to calcium binding sites; and three amino acids involved in catalysis: Lys_107_, Lys_129_ and Arg_132_. They correspond to Asp_90_, Glu_110_ and Asp_84_, as well as Lys_134_, Lys_156_ and Arg_159_, respectively (Additional file 1: Figure S3). In the PL9 family, only the crystal structure of PGL Pel9A (PDB: 1RU4) has been resolved (Jenkins et al. 2004). The three Ca^2+^ binding sites are Asp_209_, Asp_233_ and Asp_237_, and the catalytic base is Lys_273_. Based on the sequence alignment, it revealed that the above four amino acids are highly conserved in the PL9 family (Additional file 1: Figure S4). In addition, ADM99100.1 has 97% sequence identity with AAF05308.1, and AAA99476.1 shares 95% sequence identity with ADM99100.1.

In the PL10 family, there are few reports about PGLs. Using Pel10A (PDB: 1GXM) as a template, the alignment showed that the sequence identity between PGLs in the PL10 family was 25–37%. Notably, Asp_451_ is the Ca^2+^ binding site of Pel10A, Arg_524_ is a hypothetical catalytic base located at the + 1 subsite, and Glu_535_ plays a role in maintaining the structural integrity of the catalytic center group (Charnock et al. 2002). As shown in Additional file 1: Figure S5, the above three amino acids are also conserved in the PL10 family, which are likely to play a role similarly to Pel10A.

## Biochemical properties and action modes of PGLs and PMGLs

We summarized the characteristics of PGLs and PMGLs with details shown in Table 2. At present, the characterized PGLs and PMGLs are mostly derived from microorganisms such as bacteria and fungi. Notably, Ca^2+^ is essential for the activity of most PGLs, and the optimal concentrations are generally 0.5–1.0 mM (Wu et al. 2020). Of course, the optimal Ca^2+^ concentration of several PGLs is lower than 0.5 mM or higher than 1.0 mM. For instance, PGL from *Aspergillus luchuensis* var. saitoi has an optimal Ca^2+^ concentration of 0.3 mM (Kamijo et al. 2019). The activity of PpPel10 was significantly improved in 2.5 mM Ca^2+^ (Yan et al. 2018). As an activator, Ca^2+^ can acidify the C5 proton of galacturonic acid binding to the + 1 subsite of PGLs (Liu, Huo, Dai, & Dang, 2018). Therefore, Ca^2+^ plays a vital role in binding substrate, which also contributes to neutralizing the negatively charged uronic acid derived from the substrate and stabilizing the enol anion intermediate by resonance (Yang et al. 2020). Although Ca^2+^ is quite important for the activity, there are still several PGLs show activity without the presence of Ca^2+^. Li et al. found that Ca^2+^was not required for activity on pectic substrates of a highly active alkaline PGL from *Alkaliphilic Bacillus* sp. N16-5 (Gang et al. 2010). However, as shown in Table 2, the activity of PMGLs seems does not require Ca^2+^ for which their substrate is neutral pectin.

So far, a great number of PGLs and PMGLs have been characterized. It is reported that most acidic and neutral pectinases are polygalacturonase and PMGLs, and there are also some alkaline PMGLs (Anand et al. 2017). However, most PGLs have an optimal pH ranging from 8.0 to 11.5, and very few PGLs are acidic and neutral (Table 2) (Wu et al. 2020). For example, Junya et al. characterized PGL (AsPelA) with a molecular weight of 35 kDa, and its optimum pH and temperature were 8.0 and 20–40 °C (Kamijo et al. 2019). Yang et al. investigated a PGL (ApPel1) from *Aspergillus parasiticus* with an optimum pH of 4.0, it can maintain more than 60% relative activity at pH 3.0 (Yang et al. 2020). Yadav et al. purified and characterized an acidic PMGL from *Aspergillus ficuum* strain MTCC 7591 suitable for clarification of fruit juices (S. Yadav et al. 2008a, b). Sangeeta et al*.* purified and characterized the alkaline PMGL (PNL) from *Aspergillus flavus*, whose optimal pH and temperature were 8.0 and 50 °C, respectively (S. Yadav et al. 2008a, b).

Both metal ions and chemical reagents can affect the enzyme activity of PGLs and PMGLs. Some metal ions can promote the activity of one enzyme, but they may have significant inhibitory effects on another enzyme. In the presence of 5 mM divalent ions Mn^2+^ and Mg^2+^, AsPelA can retain more than 80% of its original activity, but maintains only 60% in the presence of 5 mM Cu^2+^ (Kamijo et al. 2019). After the treatment with 0.5% surfactants (SDS, TritonX-100 and Tween-20), AsPelA retains more than 90% of its original activity. However, these metal ions have significantly diverse effects on ApPel (Yang et al. 2020). Mn^2+^, Cu^2+^, Mg^2+^ and Tween-20 can activate ApPel1 with a concentration of 1 mM or 1%. The monovalent ions such as Ag^+^ and K^+^ can hardly affect the activity of ApPel1. It is worth noting that 143 mM β-mercaptoethanol (β-ME) can significantly inhibit the activity of ApPel1. Moreover, research has been conducted by adding transition metals after EDTA inhibits the activity, and it is found that YePL2B from *Yersinia enterocolitica* has the highest activity recovery in the presence of Mn^2+^ (Abbott and Boraston 2007).

Moreover, the product distribution of PGLs has been widely studied to determine the mode of action, while very few of PMGLs (Table 2). Exo-acting PGLs usually produce only monomers or dimers, endo-acting PGLs generate larger oligomers; PMGL only degrades the substrate in an endo-type manner (Bekli et al. 2019; Bonnin et al. 2009). Thus, PGLs and PMGLs have great potential in producing monosaccharides and oligosaccharides with different degrees of polymerization. It is necessary to conduct more in-depth research on the mode of action, which is conducive to the full application of pectin molecules in more fields. At present, many researchers have determined the mode of action of PGLs. For example, D. Wade Abbott and Boraston explored the PGL from *Yersinia enterocolitica*. They found that YePL2A generated saturated or unsaturated oligogalacturonic acid with DP2-5 through endo-mode of action (Abbott et al., 2007). However, YePL2B almost only produced unsaturated digalacturonic acid, indicating its exo-acting mode of activity. To analyze the action pattern of PGL (PelB) on polygalacturonic acid (PGA), Wang et al. investigated the final product by HPLC-TOF–MS (H. Wang et al. 2014). The result showed major intensive peaks at 351, 527, 703 and 879 *m*/*z*, indicating that the final product is mainly dimers, trimers, and tetramers of unsaturated galacturonic acid (GalpA). Therefore, it does not produce any monomers, which demonstrates that it is a typical endo-acting mode.

## Three-dimensional structures and catalytic mechanism of pectinolytic lyases

Diverse amino sequences contribute to various structures of PGLs and PMGLs, and the function of enzymes are closely related with the structure (Wu et al. 2020). As shown in Table 3, it showed the distribution of PGL (EC 4.2.2.2), exo-PGL (EC 4.2.2.9) and PMGL (EC 4.2.2.10) in different PL families and the corresponding structure. So far, numerous pectinolytic lyases have been characterized, but there are only partial PGLs and PMGLs have been solved crystal structure. In terms of three-dimensional structure, the current data of CAZy database shows that the PGLs of PL1, 3, and 9 families have parallel β-helix structure, the PGLs of PL2 family display (α/α)_7_-barrel structure, and the PGLs of PL10 family exhibit (α/α)_3_-barrel structure (Vincent et al. 2013). Notably, only two structures of PMGLs from PL1 family have been solved (PDB: 1IDJ and 1QCX), which show β-helix structure. This review will briefly analyze three crystal structures to understand their impact on the function of pectinase at structural level.

**Table 3:** Three-dimensional structures of PGLs and PMGLs in different PL families

PL families	Category	Primary structure
PL1 PL2 PL3 PL9 PL10	Exo-PGL (EC 4.2.2.9), PGL (EC 4.2.2.2), PMGL (EC 4.2.2.10) Exo-PGL (EC 4.2.2.9), PGL (EC 4.2.2.2), PGL (EC 4.2.2.2) Exo-PGL (EC 4.2.2.9), PGL (EC 4.2.2.2) PGL (EC 4.2.2.2)	β-helix (α/α)_7_-barrel β-helix β-helix (α/α)_3_-barrel

### β-helix structure

In the PL1, 3, and 9 families, PGLs have typical β-helix structures; two structure-solved PMGLs of PL1 family also showed β-helix structure (Vincent et al. 2013; Z. Zhou et al. 2017a, b). Specifically, the β-helix is mainly composed of three parallel β-strands (PB1, PB2, and PB3), and these β-strands are connected by three turns (Turn 1, Turn 2, Turn 3) without obvious secondary structure. The T1 turn connecting PB1 and PB2 is shorter, and the T2 and T3 turns are longer loops extending from the β-strands, which connect PB2 and PB3, PB3 and PB1, respectively (Z. Zhou et al. 2017a, b). The loops extending from the β-helix core fold on the surface and contribute to PGLs a unique shape and charge. Notably, the differences between various PGLs are the number, size and conformation of the loops protruding and covering from the parallel β-helix core (Zheng et al. 2012). According to the sequence alignment and site-directed mutagenesis studies, these protruding loops constitute the pectolytic active site (Kita et al. 1996).

**Fig. 3:**
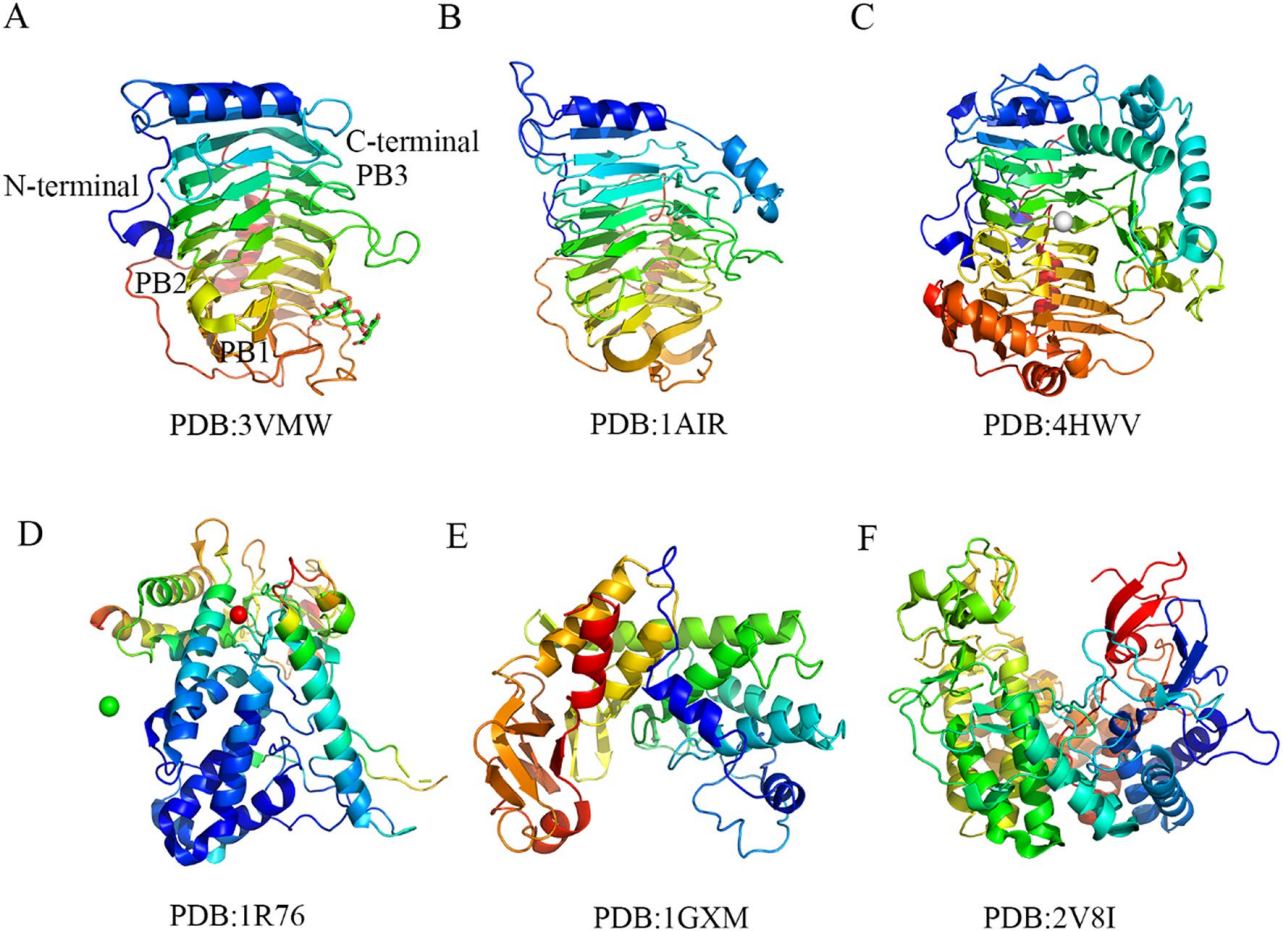
Three-dimensional structures of PGLs from different PLs families. **A**–**C**. The PGL Bsp165PelA, PelE and AcPel from the PL1 family all display typical β-helical structure. The β sheets (PB1, PB2, PB3), N-terminal and C-terminal of Bsp165PelA are marked in Fig. 3A. The white spheres in Fig. 3C are calcium ion. **D**, **E** The PGLPelA and Pel10Acm from the PL10 family are (α-α)_3_-barrel structures. The green and white spheres in Fig. 3D are chlorine and mercury ions, respectively. **F** The PL 2 family PGL YePL2A from *Yersinia enterocolitica* presents a rare (α-α)_7_-barrel structure; the corresponding PDB is marked below each structure diagram

In particular, Bsp165PelA (PDB: 3VMW) from *Bacillus* sp. N16-5 shows similar characteristics to other PGLs of the PL1 family, with parallel β-helical structure, active site residues and substrate binding clefts (Zheng et al. 2012). It appears as a parallel β-helix with 7 complete loops in the tertiary structure (Fig. 3A). The most obvious difference from other PGLs is the long T3 loop extending outward from the core structure. The turns T3 and T1 of Bsp165PelA have almost no similarity in structure. Moreover, PelE (PDB: 1AIR) from *Erwinia chrysanfhemi *can also has a parallel β-helix structure (Fig. 3B) (Lietzke et al. 1996). It forms a long loop of 26 amino acids in the N-terminal region and folds along one side of the parallel β-helix to protect the protein from erosion by solvents. At the C-terminal, the polypeptide folds into three different loops, one of which is mainly responsible for covering and protecting the C-terminal from solvent erosion. The three β strands in the structure consist of 6 to 10 β sheets, each of which has 2 to 5 residues. Similarly, AcPel (PDB: 4HWV) is a PGL of PL1 family from *Acidovor axcitrulli* and has typical right-handed β-helical fold (Tang et al. 2013). The overall structure of AcPel contains two domains, a typical right-handed β-helix consisting of a cap domain and a Ca^2+^ (Fig. 3C). Although the T2 turn is short, it is also conservative and orderly. T3 turn is longer than T1 or T2, and it participates in the formation of cap domain and active site. The acidic residues located inside the β-helix have the same side chains, pointing to the inside to stabilize the structure. The primary Ca^2+^ is located between the sheet PB1 and the turn T3, which is closely related to the substrate binding site.

### (α-α)_3_-barrel structure

Different from the β-helix structure widely existing in the PL1, 3 and 9 families, the PGLs from PL10 family exhibit (α-α)_3_-barrel structure. At present, only two crystal structures of PGLs have been solved as (α-α)_3_-barrel structure in the PL10 family, including PelA (PDB: 1R76) from *Azospirillum irakense* (Novoa de Armas et al. 2004) and Pel10Acm (PDB: 1GXM) from *Cellvibrio japonicus* (Fig. 3D, E) (Charnock et al. 2002). It provides vital information for further exploration of their structures.

To be specific, PelA displays a toroidal fold, in which the α-helical structure occupies the predominant position and is accompanied by irregular coils and short β-strands. The 384 amino acid residues of PelA are distributed in 11 anti-parallel β-strands (6%), 11 helices (38.8%) and six 3_10_ helices (5.5%). The remaining 49.7% of the residues are located in the loops and coils (Novoa de Armas et al. 2004). The structure of PelA shows two domains with a wide-open central groove for interface. According to the reports of other endo-acting polysaccharide lyases (such as xylanase), this groove is believed to carry the substrate binding site and the catalytically active site. Similarly, the active site of Pel10A cm is also located in a buried wide-open central groove between the two domains of the protein (Charnock et al. 2002). In addition, the N-terminal domain of PelA starts with a short β-strand and random coils, followed by some helical structures, β-turns and the largest α-helix. The first two helices pass through the last helix of the α loop (helix domain) mainly composed of α-helices showing a compact (α/α)_3_-barrel shape. The C-terminal domain mainly includes short β chains, irregular loops and terminal α-helices. It is more flexible than the N-terminal domain with a higher B factor.

### (α-α)_7_-barrel structure

So far, there are few reports on the (α-α)_7_-barrel structure of PGLs. In the PL2 family, PGL (YePL2A) from *Yersinia enterocolitica* exhibits an (α-α)_7_-barrel structure (Fig. 3F), which is the first discovery in prokaryotic proteins (Abbott et al., 2007). Specifically, the (α-α)_7_-barrel structure refers to packaging two anti-parallel α-helices into seven substructures. The length of each helical structure is between 10 and 20 amino acids, and these substructures are arranged in a counterclockwise direction to form the core of the barrel structure.

Interestingly, this α-barrel serves as a similar structural platform in which two large arms that are “grafted” can harness the catalytic machinery. These arms are mainly composed of β chains, which not only define the wall of the active site channel, but also give the enzyme an overall “vice-like” shape. The distance between the two arms is 15 Å with a length of 50 Å, which is long enough to hold the oligogalacturonic acid substrate (Abbott and Boraston 2007). Furthermore, the arms of the enzyme have sufficient flexibility since both the ends of the channel are generally open and when the enzyme binds to the trigalacturonic acid substrate, these arms can be closed around the substrate to fully locate the catalytic center and perform β elimination reaction.

### The catalytic mechanism of PGLs and PMGLs

PGLs and PMGLs can break the α-1,4-glycosidic bond of pectin molecule skeleton through β-elimination reaction. The β-elimination process occurs in three steps: (1) neutralization of the C-5carboxyl group that lowers the p*K*a of the H-5 proton and increases its susceptibility for abstraction; (2) proton abstraction leading to an emulate intermediate, and (3) elimination of the glycosidic bond with concomitant electron transfer from the carboxylate group to form a double bond between C-4 and C-5 (Garron and Cygler 2010). According to the acid–base proton theory, the catalytic base extracts the α-proton from the C5 atom of the acidified substrate, and the catalytic acid provides the proton to the carbonyl group (Ma et al. 2016).

**Fig. 4:**
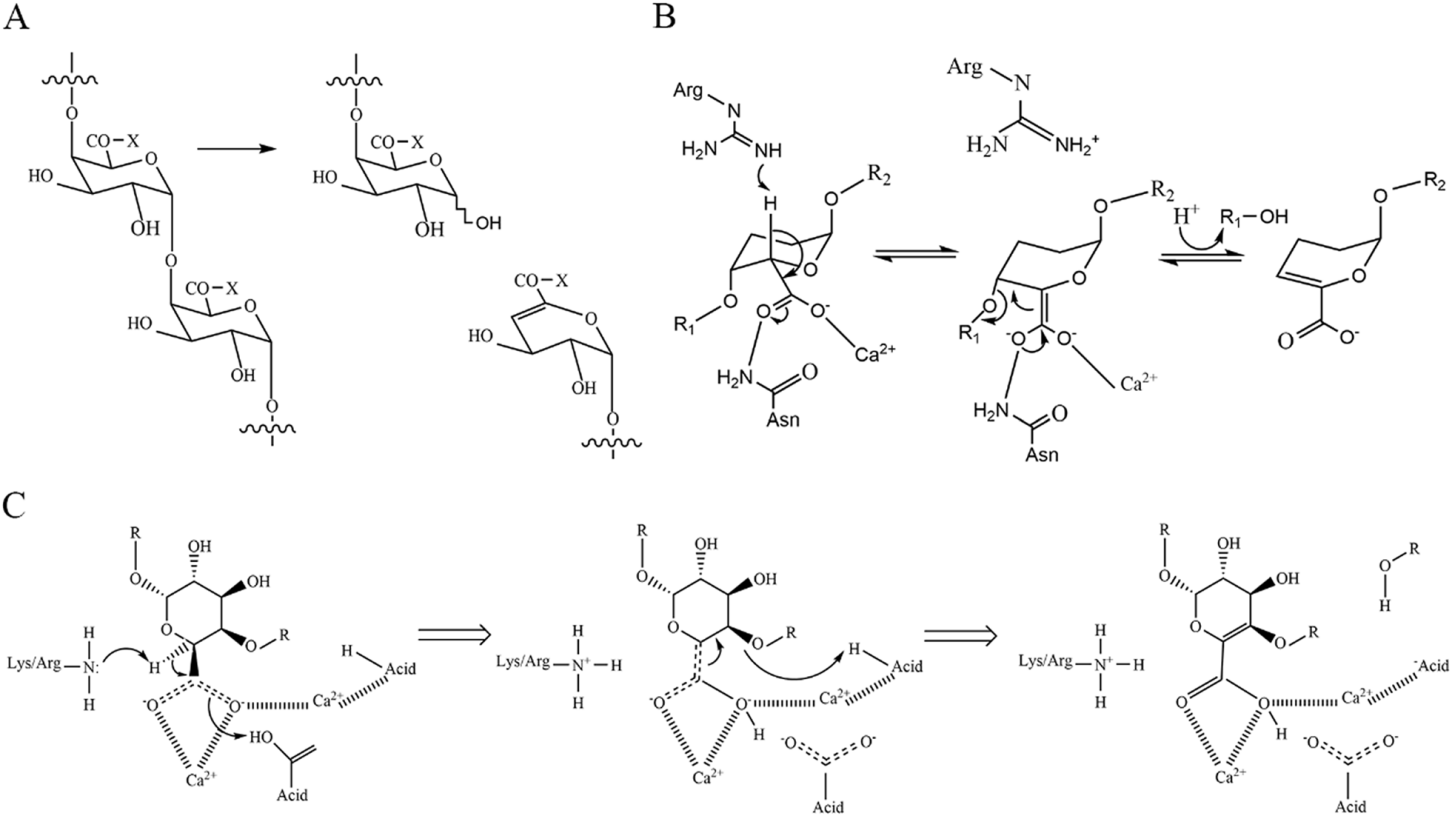
The β-elimination catalytic mechanism of PGLs. A. Mode of action in PGLs and PMGLs (X: –O–, the substrate is pectinate, X: –O–CH3, the substrate is pectin); B. Ca^2+^-assisted β-elimination catalytic mechanism; C. Common β-elimination catalytic mechanism in PGLs and PMGLs (Lys/Arg as catalytic base)

To be specific, PGLs and PMGLs attack the glycosidic bond on the side adjacent to the carboxyl group or the esterified carboxyl group. That is, the glycosidic bond is broken at the C-4 position. Meanwhile, an H atom is eliminated at the C-5 position with unsaturated C-4 and C-5 bonds formed at the non-reducing end of the newly formed oligogalacturonic acid (Fig. 4A) (Michener et al. 2015; Tang et al. 2013). Moreover, the general catalytic mechanisms have been observed among PGLs: metal-assisted neutralization of the acidic group of the sugar next to the cleaved bond, with, rather unusually, arginine or lysine playing the role of Brønsted base (Garron et al. 2010). The function of a Brønsted base is usually adopted by an arginine or alanine residue and the Brønsted acid is frequently a water molecule (Table 4). In view that the metal ions can assist β-elimination, the catalytic mechanism of PGLs can be roughly divided into Ca^2+^-assisted (Fig. 4B) and transition metal-assisted. Additionally, it can be divided into Lys/Arg-assisted β-elimination catalytic mechanism based on the different catalytic bases of PGLs and PMGLs (Fig. 4C).

**Table 4:** The catalytic mechanism of partial PMGL and PGLs

Enzyme	PDB	Neutralizer	Base	Acid	Structure	Reference
PGL	2EWE	Ca^2+^	Arg218	H_2_O	β-helix	Scavetta et al. (1999)
PMGL	1QCX	Arg176	Arg236	H_2_O	(α/α)_7_-barrel	Vitali et al. (1998)
PGL	2V8K	Mn^2+^	Arg171	H_2_O	β-helix	Abbott and Boraston (2007)
PGL	3B8Y	Ca^2+^	Lys224	H_2_O	β-helix	Creze et al. (2008)
PGL	1RU4	Ca^2+^	Lys273	H_2_O	β-helix	Jenkins et al. (2004)
PGL	1GXO	Ca^2+^	Arg524	H_2_O	(α/α)_3_-barrel	Charnock et al. (2002)

### Ca^2+^-assisted β-elimination reaction

The catalytic mechanism of PGLs is typical β-elimination process. At present, many studies have shown that Ca^2+^ can effectively assist the β-elimination reactions (Charnock et al. 2002; C. Creze et al. 2008; Jenkins et al. 2004; R. D. Scavetta et al. 1999). Of them, Lys/Arg acts as Brønsted base and water molecule as a Brønsted acid (Table 4). Most PGLs require Ca^2+^ for their catalytic activity, because it can acidify substrate at the active site. There are two types of Ca^2+^ in PGLs, primary Ca^2+^ and additional Ca^2+^. The former binds to enzyme molecules without a substrate, the latter acts as the bridge between enzymes and the oligogalacturonic acid complex (Scavetta 1999; Seyedarabi et al. 2010; Zheng et al. 2012). In the enzyme–Ca^2+^–galacturonic acid complex, electrostatic interaction plays a dominant role. The negatively charged part of galacturonate mainly interacts with Ca^2+^ or positively charged groups in proteins (Zheng et al. 2012). As shown in Fig. 4B, it illustrates the calcium-assisted β-elimination mechanism.

### Transition metal-assisted β-elimination reaction

Except for Ca^2+^, there are other transition metals which assisted β-elimination reaction, such as Mn^2+^ or Ni^2+^. In the PL2 family, The PGL (YePL2A) from *Yersinia enterocolitica* adopts rare (α/α)_7_-barrel structure and unique transition metal-assisted β-elimination (Abbott and Boraston 2007). YePL2A has two characteristics: (1) it uses metal atoms other than calcium for catalysis; (2) its Brønsted base presents an alternate conformation, which can directly interact with the uronic acid group of the substrate. Interestingly, the putative catalysis (Arg171) in YePL2A approaches the substrate from the opposite direction to the PGLs of the PL1 and 10 families. In this conformation, the imino nitrogen forms a new interaction with the uronic acid group of the substrate, which may help stabilize the reaction intermediate. Considering the biochemical and structural data, it is strongly supported that the transition metal Mn^2+^ or Ni^2+^ in YePL2A assists the β-elimination mechanism. However, more details about the mechanism of transition metal-assisted β-elimination still need to be further explored.

### β-Elimination reaction with Arg as a catalytic base

Based on the residues playing the role of Brønsted base and acid, the catalytic mechanisms fall into two categories: (1) Lys as Brønsted base and water molecule as a Brønsted acid; (2) Arg as Brønsted base and water molecule as a Brønsted acid. In the PL1 family, Ma et al. suggested that the PGL (BsPel) from *Bacillus subtilis* belongs to the second case (Ma et al. 2016). As a catalytic base, Arg279 can extract α-protons from the C_52_ atom of the substrate subsite, thereby forming an unstable carbanion intermediate (Fig. 4C). Three Ca^2+^ ions in the active site play a vital role in tightly binding the substrate through coordination interactions. Therefore, Ca2 and Ca3 can promote proton transfer by acidifying α-protons and stabilize the intermediates produced by the reaction. Lys247 and Arg279 are both prerequisites for promoting catalysis by forming hydrogen bonds with the C_52_-Coo^−^ of the substrate. In addition, quantum mechanics and molecular methods (QM/MM) calculations also confirmed that the source of protons for the leaving group is the solvent water molecule, namely catalytic acid.

### β-Elimination reaction with Lys as a catalytic base

There are some PGLs utilizing lysine as Brønsted base instead of arginine (C. Creze et al. 2008; Jenkins et al. 2004). For example, Markus et al. investigated the PGL of PL3 family from *Caldicellulosiruptor bescii* and revealed that Lys108 acted as a catalytic base in the β-elimination reaction, without clear catalytic acid candidates (Michener et al. 2015). The reaction mechanism can be explained by the anti-planar trans-elimination reaction, in which Lys108 extracts protons from C5 atoms without providing protons through acidic residues. The acidified water molecule completes the trans β-elimination reaction by prorogating the O4 atom of the substrate. For galacturonic acid, the C5 hydrogen and C4 hydroxyl of the substrate must be oriented in an axial configuration to be possible. In addition, the acidic residues Glu84, Glu39 and Asp107 are involved in calcium coordination: the active center has three Ca atoms, and Glu84 is necessary for the binding of two Ca atoms. According to structural data, Gln111 and Arg133 play a role in the substrate binding. Arg133 is particularly important for the correct positioning of the substrate; and Gln111 has little effect on the substrate binding, it seems to stabilize the O4 atom in the last step of the reaction.

As previously discussed, there is still insufficient information regarding the structural and catalytic mechanisms of PGLs and PMGLs because only partial enzyme structures have been solved. Therefore, we only classified and summarized the structures and catalytic mechanisms that have been settled, and illustrated them with examples. However, with the development of advanced technologies, such as high-throughput sequencing technology and bioinformatics, it is feasible to obtain insights into the structures and catalytic mechanisms of these enzymes in the future.

## Conclusion

PGLs and PMGLs have been favored by many researchers, because they efficiently degrade pectin through β-elimination mechanism without producing highly toxic methanol. This review clarifies the substrate, source, classification, characteristics, and action pattern of PGLs and PMGLs. It provides information about sequence alignment and three-dimensional structure. PGLs from PL1, 3 and 9 families have parallel β-helical structures, the PL2 family PGLs display rare (α/α)_7_-barrel structure, and the PGLs from PL10 family show (α/α)_3_-barrel structure. Moreover, PMGLs have β-helix structure in the PL1 family.

PGLs and PMGLs can cleave the α-1,4-glycosidic bond of substrate molecules by trans β-elimination reactions. Considering the different metal ions that assist β-elimination, the catalytic mechanism can be roughly divided into Ca^2+^ and transition metal ion-assisted types. According to the catalytic base, it can be classified as Lys/Arg type β-elimination catalytic mechanism. In conclusion, PGLs and PMGLs have significant roles in various biotechnological applications, such as textile industry, paper making and juice clarification. Further investigation on these enzymes could result in full utilization of substrate resources and better commercial implications.

### Supplementary Information

**Additional file 1:** Supplementary material to Bioresources and Bioprocessing. **Fig. S1.** Multiple sequences alignment of PGLs from PL1 family. The sequences of PGLs aligned were listed as follows: CAB12585.1 from *Bacillus subtilis*; AAE59748.1 from *Alkalihalobacillu shalodurans*; BAB07538.1 from *Bacillus halodurans* C-125; CAD56882.1 from *Bacillus licheniformis* 14A; BAB04417.1 from *Bacillus halodurans* C-125. **Fig. S2.** Multiple sequences alignment of PGLs from PL2 family. The sequences of PGLs aligned were listed as follows: CAL14085.1 from* Yersinia enterocolitica* 8081; AJI84205.1 from* Yersinia enterocolitica*; AAA27660.1 from* Yersinia pseudotuberculosis*; AAA24851.1 from* Pectobacterium carotovorum* EC153; CAA34432.1 from* Pectobacterium carotovorum* SCRI193. **Fig. S3.** Multiple sequences alignment of PGLs from PL3 family. The sequences of PGLs aligned were listed as follows: BAA87892.1 from* Bacillus* sp. KSM-P15; ADB78744.1 from* Paenibacillus amylolyticus* 27C64; ACM60942.1 from* Caldicellulosiruptor bescii* DSM 6725; AAA57140.1 from* Pectobacterium carotovorum*; ADM99410.1 from* Dickeyadadantii* 3937. **Fig. S4.** Multiple sequences alignment of PGLs from PL9 family. The sequences of PGLs aligned were listed as follows: ADM99100.1 from* Dickeyadadantii* 3937; AAF05308.1 from* Dickeyachrysanthemi* PY35; AAA99476.1 from* Dickeyachrysanthemi* EC16; ADO59170.1 from *Paenibacillus polymyxa* SC2; AAK79928.1 from *Clostridium acetobutylicum* ATCC 824. **Fig. S5.** Multiple sequences alignment of PGLs from PL10 family. The sequences of PGLs aligned were listed as follows: ACE85516.1 from *Cellvibrio japonicus* Ueda107; BAA81752.1 from *Bacillus* sp. KSM-P15; AAG24437.1 from *Alkalihalobacillus alcalophilus* NTT33; AAD25394.1 from *Niveispirillumirakense* KBC1; AFQ23188.1 from *Xanthomonas campestris* ACCC 1004.

## Data Availability

All data supporting the findings of this study are available in the article, supporting information, or upon request from the corresponding author.
