# Supplementary material for: Pectinolytic lyases: a comprehensive review of sources, category, property, structure, and catalytic mechanism of pectate lyases and pectin lyases
Source: Bioresour Bioprocess. 2021 Aug 23;8(1):79. doi: 10.1186/s40643-021-00432-z (PMC10992409; doi:10.1186/s40643-021-00432-z)
Supplement: Supplementary file 1 — Additional file 1: Supplementary material to Bioresources and Bioprocessing. Fig. S1. Multiple sequences alignment of PGLs from PL1 family. The sequences of PGLs aligned were listed as follows: CAB12585.1 from Bacillus subtilis; AAE59748.1 from Alkalihalobacillu shalodurans; BAB07538.1 from Bacillus halodurans C-125; CAD56882.1 from Bacillus licheniformis 14A; BAB04417.1 from Bacillus halodurans C-125. Fig. S2. Multiple sequences alignment of PGLs from PL2 family. The sequences of PGLs aligned were listed as follows: CAL14085.1 from Yersinia enterocolitica 8081; AJI84205.1 from Yersinia enterocolitica; AAA27660.1 from Yersinia pseudotuberculosis; AAA24851.1 from Pectobacterium carotovorum EC153; CAA34432.1 from Pectobacterium carotovorum SCRI193. Fig. S3. Multiple sequences alignment of PGLs from PL3 family. The sequences of PGLs aligned were listed as follows: BAA87892.1 from Bacillus sp. KSM-P15; ADB78744.1 from Paenibacillus amylolyticus 27C64; ACM60942.1 from Caldicellulosiruptor bescii DSM 6725; AAA57140.1 from Pectobacterium carotovorum; ADM99410.1 from Dickeyadadantii 3937. Fig. S4. Multiple sequences alignment of PGLs from PL9 family. The sequences of PGLs aligned were listed as follows: ADM99100.1 from Dickeyadadantii 3937; AAF05308.1 from Dickeyachrysanthemi PY35; AAA99476.1 from Dickeyachrysanthemi EC16; ADO59170.1 from Paenibacillus polymyxa SC2; AAK79928.1 from Clostridium acetobutylicum ATCC 824. Fig. S5. Multiple sequences alignment of PGLs from PL10 family. The sequences of PGLs aligned were listed as follows: ACE85516.1 from Cellvibrio japonicus Ueda107; BAA81752.1 from Bacillus sp. KSM-P15; AAG24437.1 from Alkalihalobacillus alcalophilus NTT33; AAD25394.1 from Niveispirillumirakense KBC1; AFQ23188.1 from Xanthomonas campestris ACCC 1004. [file 40643_2021_432_MOESM1_ESM.docx]

Supplementary material to **Applied Microbiology and Biotechnology**

**Pectinolytic lyases: A comprehensive review of sources, category, property, structure, and catalytic mechanism of pectate lyases and pectin lyases**

Ling Zheng^1^, Yin-xiao Xu^1^, Qian Li^1^, Ben-wei Zhu^1,*^

^1^College of Food Science and Light Industry, Nanjing Tech University, Nanjing 211816, China

^*^ Corresponding author

Ben-wei Zhu

e-mail: zhubenwei@njtech.edu.cn

phone: +86 025-58139419 fax: +86 025-58139419


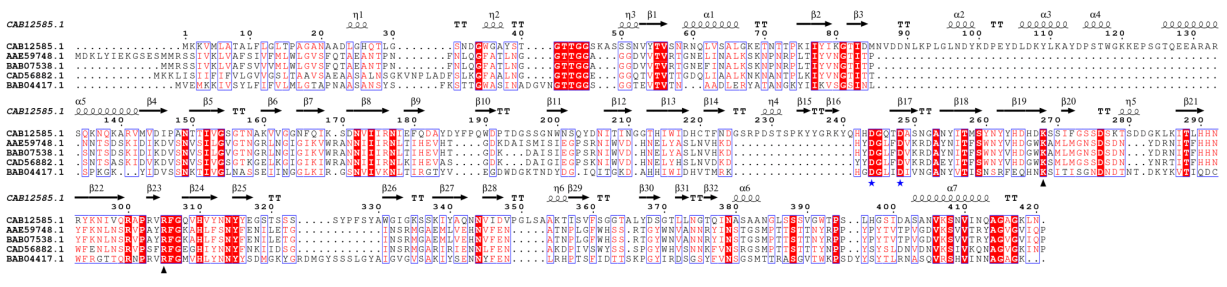


**Fig. S1 Multiple sequences alignment of PGLs from PL1 family.**

The sequences of PGLs aligned were listed as follows: CAB12585.1 from *Bacillus subtilis*; AAE59748.1 from *Alkalihalobacillu shalodurans*; BAB07538.1 from *Bacillus halodurans* C-125; CAD56882.1 from *Bacillus licheniformis* 14A; BAB04417.1 from *Bacillus halodurans* C-125.


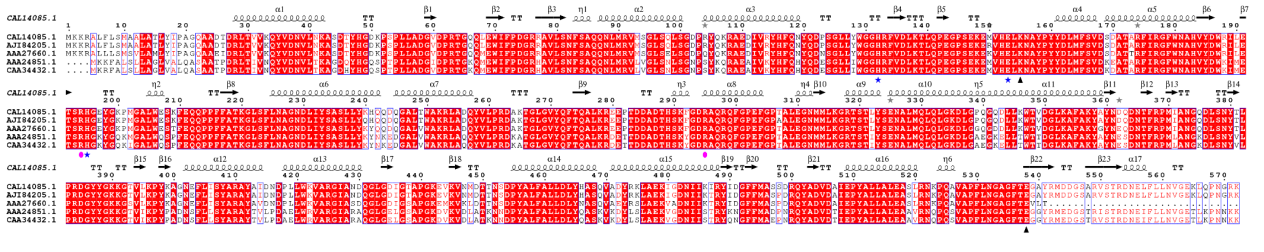


**Fig. S2 Multiple sequences alignment of PGLs from PL2 family.**

The sequences of PGLs aligned were listed as follows: CAL14085.1 from *Yersinia enterocolitica 8081*; AJI84205.1 from *Yersinia enterocolitica*; AAA27660.1 from *Yersinia pseudotuberculosis*; AAA24851.1 from *Pectobacterium carotovorum* EC153; CAA34432.1 from *Pectobacterium carotovorum* SCRI193


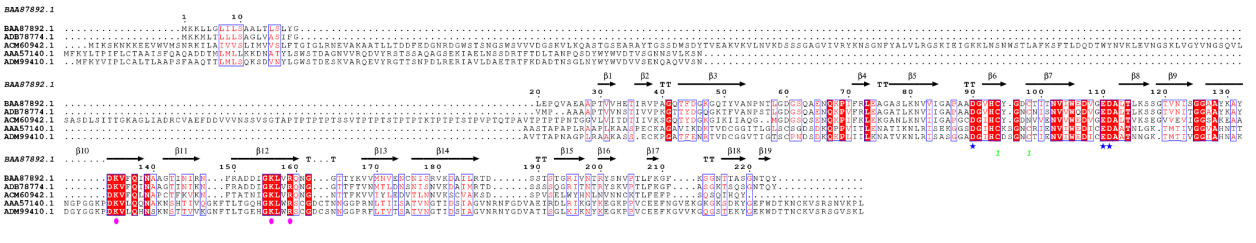


**Fig. S3 Multiple sequences alignment of PGLs from PL3 family.**

The sequences of PGLs aligned were listed as follows: BAA87892.1 from *Bacillus* sp. KSM-P15; ADB78744.1 from *Paenibacillus amylolyticus* 27C64; ACM60942.1 from *Caldicellulosiruptor bescii* DSM 6725; AAA57140.1 from *Pectobacterium carotovorum*; ADM99410.1 from *Dickeyadadantii* 3937


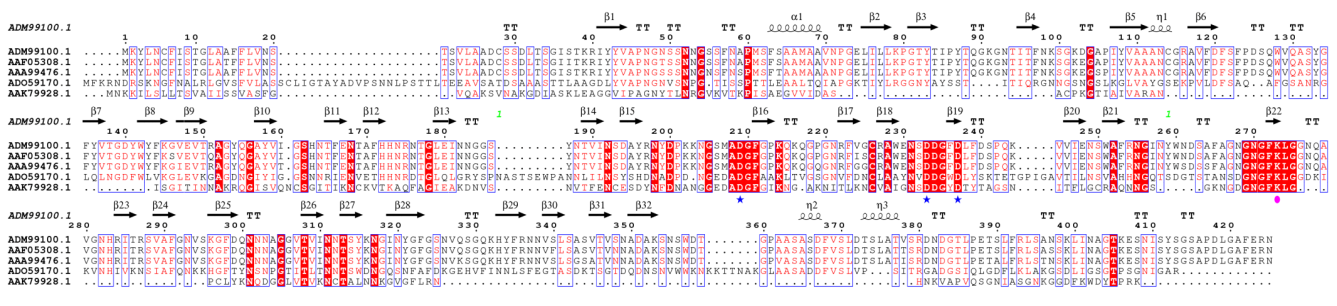


**Fig. S4 Multiple sequences alignment of PGLs from PL9 family.**

The sequences of PGLs aligned were listed as follows: ADM99100.1 from *Dickeyadadantii* 3937; AAF05308.1 from *Dickeyachrysanthemi* PY35; AAA99476.1 from *Dickeyachrysanthemi* EC16; ADO59170.1 from*Paenibacillus*

*-polymyxa* SC2; AAK79928.1 from *Clostridium acetobutylicum* ATCC 824.


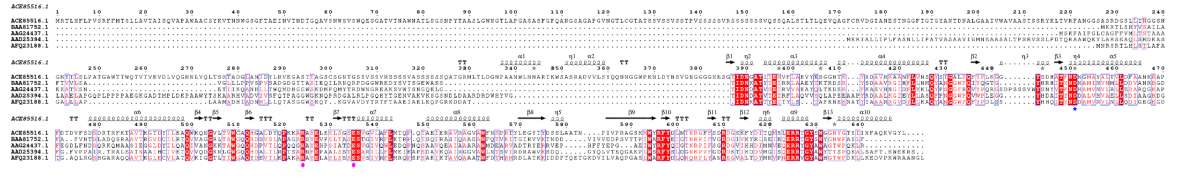


**Fig. S5 Multiple sequences alignment of PGLs from PL10 family.**

The sequences of PGLs aligned were listed as follows: ACE85516.1 from *Cellvibrio japonicus* Ueda107; BAA81752.1 from *Bacillus* sp. KSM-P15; AAG24437.1 from *Alkalihalobacillusalcalophilus* NTT33; AAD25394.1 from *Niveispirillumirakense* KBC1; AFQ23188.1 from *Xanthomonas campestris* ACCC 1004.
